# Evaluation of ST-segment agreement between Spandan Pro and gold standard electrocardiogram for percutaneous coronary intervention decision-making

**DOI:** 10.1186/s43044-025-00691-7

**Published:** 2025-10-07

**Authors:** CB Pandey, Yogendra Singh, Shashank Pandey, Deepak Tomar, Nitin Chandola, Deeksha Agarwal, Sengar Yashwardhan Pratap Singh

**Affiliations:** 1 Lala Lajpat Rai Medical College, Meerut, India; 2 Max Super Speciality Hospitals, Dehradun, India; 3 Sunfox Technologies, Dehradun, India

**Keywords:** Bland–Altman analysis, Coronary artery disease, Smartphone-based ECG, Spandan pro ECG, ST-elevation, Percutaneous coronary intervention

## Abstract

**Background:**

ST-elevation myocardial infarction (STEMI) is a critical condition requiring rapid diagnosis and treatment. Smartphone-based Electrocardiogram (ECG) devices, like Spandan Pro, offer the potential for timely Percutaneous Coronary Intervention (PCI) in STEMI patients, particularly in resource-limited environments.

**Objective:**

To assess the agreement between ST-segment elevation measurements obtained from the Spandan Pro ECG device and those from a Gold standard ECG (BPL Cardiart), using Bland–Altman (BA) analysis, in the context of decision-making for PCI.

**Methods:**

A cross-sectional, observational study was conducted on 200 patients who presented to the local Hospital with complaints of chest pain. After strict application of exclusion criteria, a total of 184 patients were assessed in the study. BA analysis has been used to estimate the agreement between ST-segment elevation measurements obtained from the Spandan Pro ECG device with the Gold standard ECG. The ECG reports assist the cardiologist in making decisions regarding PCI.

**Results:**

Of the 184 patients, 55 met the criteria for PCI, 33 of whom presented within 120 h of symptom onset. BA analysis revealed that the mean differences between the two methods were clinically insignificant, with agreement limits falling within acceptable ranges across all leads, confirming the reliability of the Spandan Pro.

**Conclusion:**

The Spandan Pro ECG device shows good agreement in diagnosing ST elevation with the Gold standard ECG, making it a valuable tool in decision-making regarding PCI by the cardiologist. It also improves patient outcomes by enabling rapid diagnosis and treatment, especially in resource-limited or prehospital environments.

## Introduction

**What’s New:**

The agreement of ST-segment interpretation between a gold-standard 12-lead ECG and smartphone-based ECG (Spandan Pro) in the context of percutaneous coronary intervention (PCI) decision-making.

In terms of ST-elevation detection, the Spandan Pro ECG shows good concordance with the Gold standard ECG, which makes it suitable for usage in remote and emergency settings to facilitate timely triage and decision-making.

The results confirm the clinical viability of mobile ECG devices for decentralized, rapid, and accurate STEMI detection, which may have implications for reducing the time to PCI.

Coronary artery disease (CAD) remains one of the leading causes of mortality worldwide, with myocardial infarction (MI) as its most serious clinical consequence [1]. Notably, around 90% of MI cases reveal obstructive CAD on angiography, highlighting the importance of early detection and prompt treatment of the disease to improve survival and reduce long-term complications [2, 3]. Among the various types of MI, ST-segment elevation myocardial infarction (STEMI) represents a major global health burden that necessitates urgent diagnosis and reperfusion therapy.

Current clinical guidelines recommend rapid acquisition of a 12-lead electrocardiogram (ECG) to confirm the diagnosis of STEMI and enable timely primary percutaneous coronary intervention (PCI), ideally within 90 min of presentation to a PCI-capable facility or within 120 min if transfer is required [4, 5]. The traditional ECGs, nevertheless, are sometimes limited to clinical environments and need trained staff as well as specialized machines [6]. These restrictions have the potential to cause delays, particularly in rural or resource-constrained settings.

**Fig. 1:**
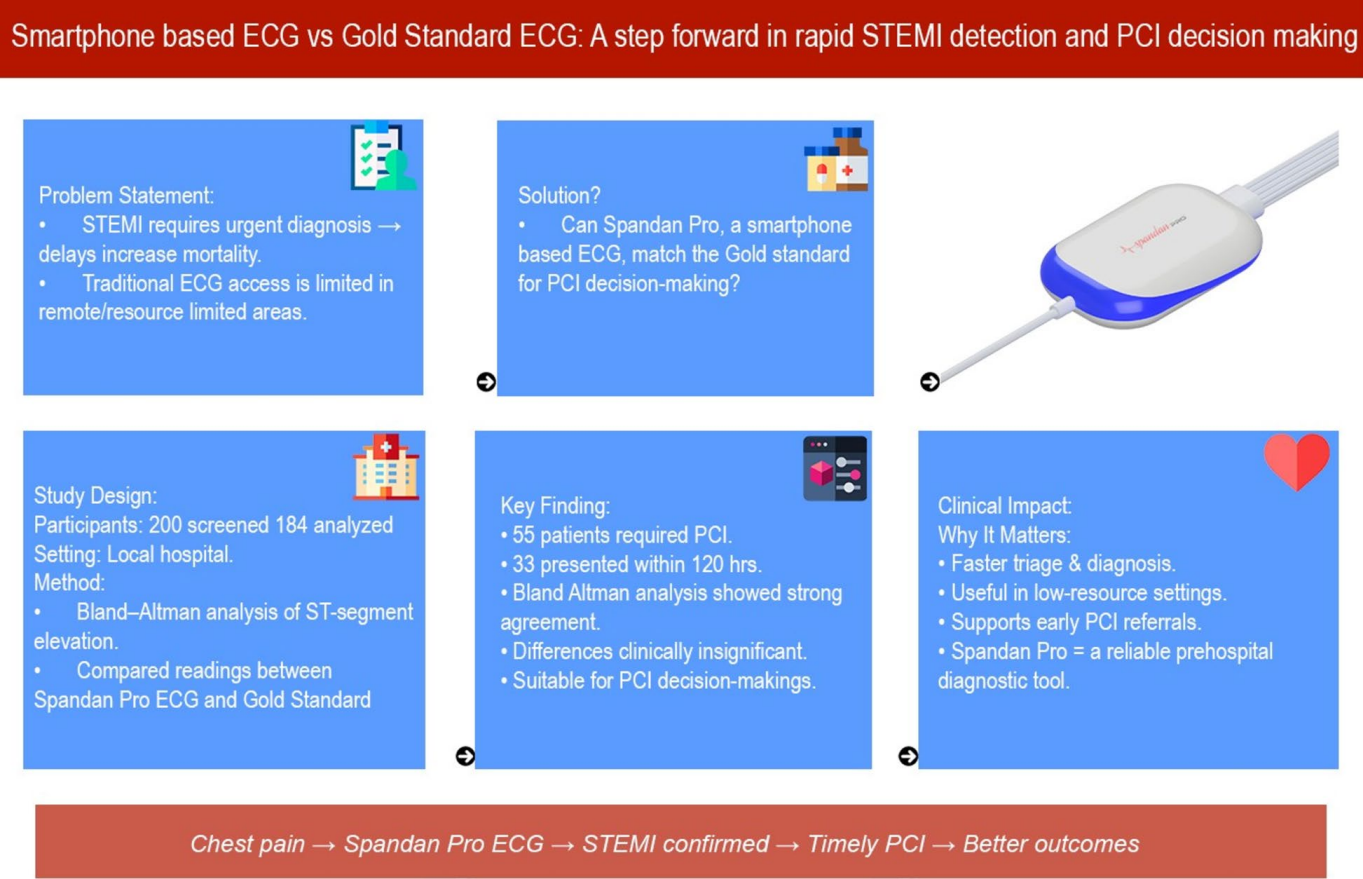
Infographic- Smartphone-based ECG vs Gold Standard ECG: A step forward in rapid STEMI detection and Percutaneous Coronary Intervention (PCI) decision-making

Smartphone-based ECG devices have emerged as innovative solutions to overcome these barriers, offering portability, real-time data acquisition, and ease of use. Among these, the Spandan Pro ECG device developed by Sunfox Technologies enables high-quality 12-lead-equivalent recordings using a smartphone interface, providing clinicians with immediate access to ECG data (As shown in Fig. 1). Prior studies have shown that Spandan and other mobile ECG devices can detect STEMI with comparable accuracy to conventional systems [7–15], and some even integrate artificial intelligence to enhance diagnostic precision [16].

Recent studies, including a case study by Singh Y et al. (2024), have proven Spandan Pro's capability in the detection of early ischemia and directing appropriate intervention on time. This has created interest in validating its role in clinical decision-making, especially for PCI [17].

Though smartphone-based ECG devices have diagnostic ability to detect STEMI, their ability to aid in PCI decision guidance has not been adequately assessed. Although the devices can detect ST-segment elevation, evidence concerning their direct role in guiding clinical decisions, like whether to immediately perform PCI, is lacking. The lack of clinical validation for such use restricts their integration into emergency care processes. This research fills that void by comparing the diagnostic accuracy of a smartphone-acquired ECG device to that of a Gold standard 12-lead ECG to identify ST-segment elevation and ascertain the necessity of PCI. The findings could confirm the device's ability to aid in quick triage and therapeutic interventions in acute cardiac care, particularly in resource-poor environments.

## Methods

### Study design

This study was designed as a cross-sectional, single-blinded, and observational study. It aimed to evaluate the agreement between ST-segment elevation measurements obtained from the Spandan Pro smartphone-based ECG device and those from a Gold standard 12-lead ECG (BPL Cardiart), in the context of decision-making for PCI.

### Participants and settings

The study cohort included 200 individuals and was conducted in the emergency and ECG rooms at the local Hospital in Meerut, from 16 May 2023 to 24 November 2023, and utilized patients who were 20 years of age or older. The sample size was estimated using Yamane’s formula:

$$n = N/\left( {1 \, + \, Ne^{2} } \right),$$

where n is the required sample size, N is the estimated population size, and e is the margin of error.

The study included 184 participants, and these participants were selected on the basis of inclusion criteria such as chest pain within or after 120 h with ST elevation more than 1 mm in two or more leads on admission ECG. The patients who were suffering from dementia, bundle branch block, cardiogenic shock, history of bleeding risk, critically ill patients, hemodynamic instability, pregnant women, and reports with poor quality ECG tracings due to baseline wandering or artifacts, or participants who refused to participate were excluded from the study.

### Supervision and oversight

All tests and procedures were done directly under the supervision of a cardiologist to ensure that they were conducted according to Gold standard protocols and in the most minimal possible source of bias.

## Reference standard

The conventional Gold standard 12-lead ECG and the Spandan Pro 12-lead ECG were compared for diagnostic accuracy. A cardiologist interpreted both ECG reports, and the interpretations were then compared to confirm that the Spandan Pro ECG device aligned with PCI recommendations. To reduce bias and increase the study's reliability and accuracy, the cardiologist was blinded to the devices, along with their computerized interpretations. The cardiologist's interpretation of the conventional gold standard 12-lead ECG was the only basis for participants' treatment decisions. The Spandan Pro ECG device could also be used to guide treatment decisions, although in this study, neither direct interventions nor treatment decisions were based on its findings.

### Data collection

Every participant who presented with chest pain was evaluated, and their informed consent was collected. The participants' case report format (CRF), which contained comprehensive information on the participants' clinical history and demographics, was then completed by trained clinical trial assistants. The sponsor has provided training to the staff regarding the trial Protocol, CRF, the Informed consent form, and product-related training. The CRF assisted the cardiologist in diagnosing the case. The first set of ECG recordings was taken with both the gold standard 12-lead ECG and the Spandan Pro smartphone-based ECG device. Decision support regarding treatment was made based on the interpretation of the gold standard ECG, while the ECG that was obtained through Spandan Pro was analyzed independently by the cardiologist to validate accuracy. Both ECG devices were calibrated as per Standard IEC 60601–2-25 (Requirements for the Basic Safety and Essential Performance).

### Timing considerations

To reduce interpretation bias in this comparative study, there was a time gap of not more than 5 to 6 h between the smartphone-based ECG recording and the gold standard 12-lead ECG recording. This time restriction was put in place to ensure uniformity in the comparison by accounting for cases in which the gold standard ECG was conducted outside of regular working hours (late night to early morning) or in emergency department cases where the physician’s primary focus is on stabilizing the serious patient, which may delay the recording of the Spandan ECG. Although a 5–6 h gap could introduce minor clinical variation, this interval was carefully restricted to minimize such effects while still enabling a rigorous comparison.

### Statistical analysis

The agreement of ST-segment elevation measurements derived from the Spandan Pro device and the gold standard ECG on which the decision to perform PCI was based was tested by Bland–Altman (BA) analysis. Data were processed with Microsoft Excel.

#### Data privacy, security, confidentiality, storage, and archival

The hospital ensures that it will never disclose information to any department, company, or third party, as the safety of patients' information and their privacy are our major concerns. The information will be handled according to highly rigorous data handling procedures. All the data will be anonymized at the point of collection and stored securely on a two-step authenticated hospital local server, with backup storage managed through HIPAA-compliant cloud storage spreadsheets and also printed sheets stored within the hospital. Access to the data will be limited to personnel from the hospital who are authorized. No personally identifiable information will be linked to the analyzed data. Furthermore, all data transfer will take place over secure, encrypted channels to ensure data integrity and confidentiality.

#### Secondary analysis of PCI decision-making trial using Spandan Pro ECG

This Study is a secondary analysis of data obtained from a previously published cross-sectional observational trial that had assessed the diagnostic utility of the Spandan Pro smartphone ECG device in decision-making for PCI in patients with suspected STEMI [18]. The initial study had 184 adult patients and showed excellent agreement between the Spandan Pro and Gold standard 12-lead ECG using paired t-tests and Pearson correlation coefficients. In the present analysis, we extend those findings by specifically assessing the agreement of ST-segment elevation measurements by BA analysis to further assess the consistency and clinical reliability of the Spandan Pro ECG machine in determining PCI decisions.

## Results

A total of 200 patient records were analyzed, with 16 cases excluded from baseline wandering. A total of 184 cases were presented for analysis. Of these, 96 patients developed chest pain within 120 h, while 88 patients developed chest pain after 120 h. 55 patients had an ST-segment elevation within or after 120 h of symptom onset, hence fitting the PCI recommendation criteria, while 129 did not. Of the 55 PCI-eligible patients, 33 were presented within the 120-h window, and 22 came after. Accuracy testing of both the Spandan Pro ECG device and the gold standard method showed perfect concordance with PCI recommendations, meaning perfect agreement with clinical guidelines.

Among 184 patients, the patients’ demographic and clinical data were divided into patients for whom PCI was recommended and those for whom the procedure was not recommended. Key variables include gender, with 136 males (45 recommended for PCI, 91 not recommended) and 48 females (10 recommended, 38 not recommended). Diabetic, 33 (8 for PCI and 25 not for PCI); smokers, 81 (28 for PCI and 53 not for PCI); CAD, 68 (32 for PCI and 36 not for PCI).

Table 1 allocates STEMI and Non-ST-elevation Myocardial Infarction (NSTEMI) diagnoses into PCI-recommended cases, which outlines the spread of MI and ischemia in various parts of the heart. It comprises 16 cases of anterior wall MI, 1 anterolateral MI, 33 anteroseptal MI, 7 inferior wall MI, 6 inferolateral MI, and 1 lateral wall MI. Ischemic cases include 6 cases of inferior-lateral ischemia, 3 cases of anterior-septum ischemia, 1 case of each for the anterior wall, inferior wall, and lateral wall ischemia, and 4 cases of unspecified ischemia. This provides a comprehensive view of the infarction and ischemia locations in patients recommended for PCI.

**Table 1:** Classification of STEMI/NSTEMI Diagnosis in PCI Recommended Cases

Parameters	Total Number of Cases
Anterior wall MI	16
Anterolateral MI	1
Anteroseptal MI	33
Inferior wall MI	7
Inferolateral MI	6
Lateral wall MI	1
Inferolateral ischemia	6
Anteroseptal ischemia	3
Anterior wall Ischemia	1
Inferior wall Ischemia	1
Lateral wall Ischemia	6
Ischemia	4

**Fig. 2:**
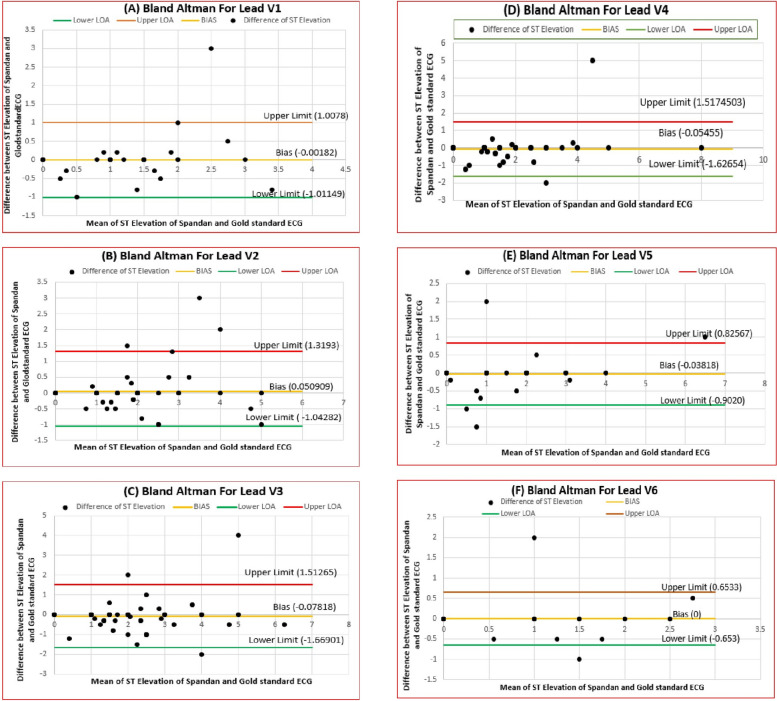
Bland–Altman plots illustrate varying levels of agreement between ST elevation measurements obtained from the Spandan Pro ECG device and the Gold Standard ECG for individual leads: (2A) Lead V1, (2B) Lead V2, (2C) Lead V3, (2D) Lead V4, (2E) Lead V5, and (2F) Lead V6

**Fig. 3:**
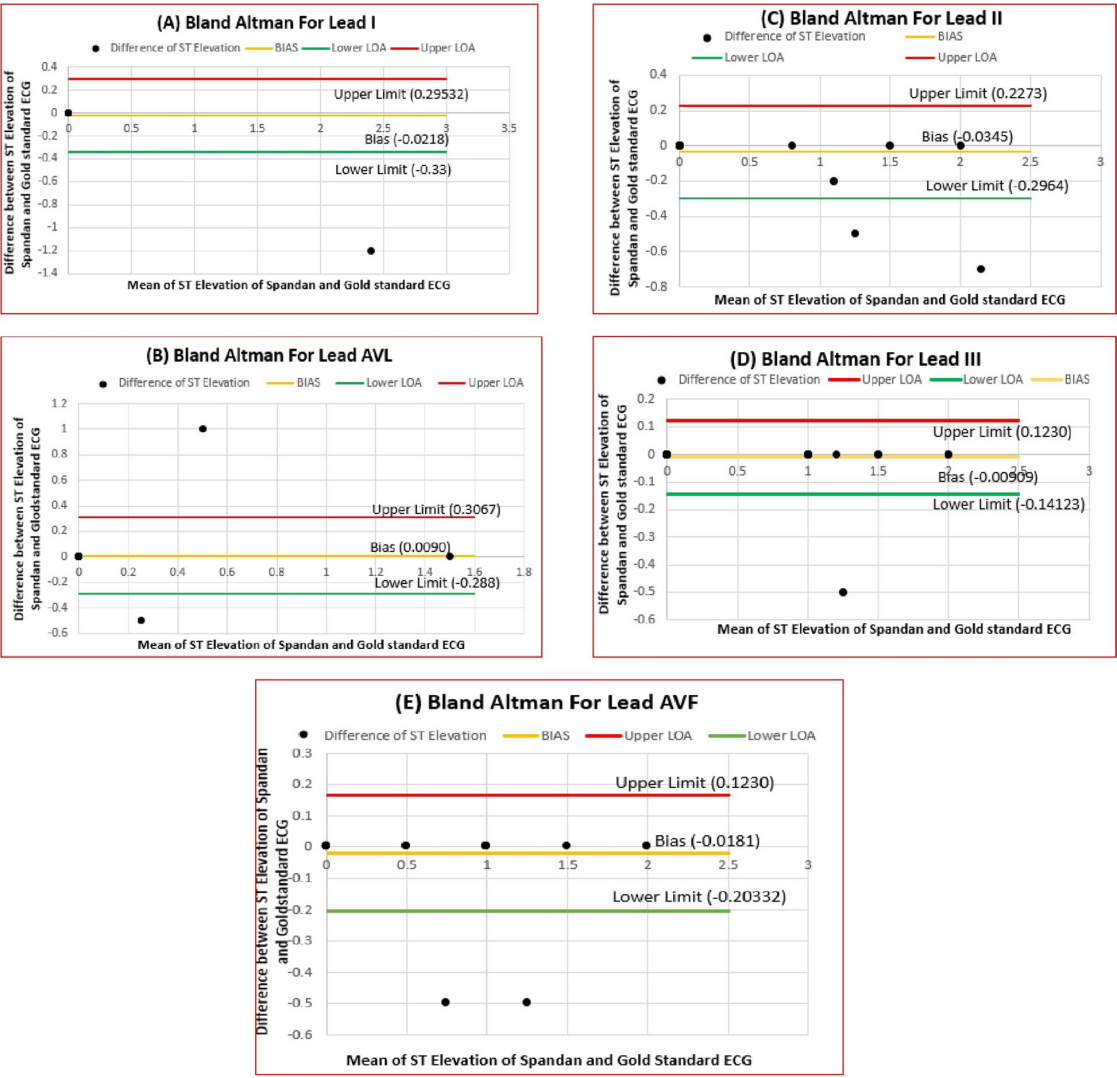
Bland–Altman plots illustrate varying levels of agreement between ST elevation measurements obtained from the Spandan Pro ECG device and the Gold Standard ECG for individual leads: (3A) Lead I, (3B) AVL, (3C) Lead II, (3D) Lead III, and (3E) Lead AVF

Table 2 indicates the BA comparison of the Spandan Pro algorithm for the ST-elevation measurements with those of a Gold standard ECG for all 12 leads. The comparison indicated excellent agreement, as the mean differences were in the range of  −0.08 mV to 0.05 mV and clinically acceptable limits of agreement. Figure 2 shows the outcome for precordial leads (V1–V6), and Fig. 3 shows the limb leads (I, II, III, aVL, aVF), which exhibit minimal bias and consistent measurements. These results validate the clinical accuracy and reliability of the Spandan Pro algorithm for ST-elevation measurement.

**Table 2:** Bland–Altman Analysis of ST-Elevation: Spandan Pro vs. Gold Standard ECG

Lead	Mean Difference (mV)	Lower LOA (mV)	Upper LOA (mV)	Range of LOA (mV)	Interpretation
V1	−0.002	−1.01	1.01	2.02	Negligible bias, high agreement
V2	0.05	−1.04	1.32	2.36	Minor bias, acceptable agreement
V3	−0.08	−1.67	1.51	3.18	Slight bias, clinically acceptable
V4	−0.05	−1.63	1.52	3.15	Minor bias, acceptable
V5	−0.04	−0.90	0.82	1.72	Narrow LOA, strong agreement
V6	0	−0.65	0.65	1.30	No bias, very high agreement
I	−0.02	−0.33	0.29	0.62	Extremely consistent
aVL	0.01	−0.29	0.31	0.60	Very accurate
II	−0.03	−0.30	0.23	0.53	Small, consistent differences
III	−0.01	−0.14	0.12	0.26	Highly accurate
aVF	−0.01	−0.20	0.16	0.36	Consistent and reliable

## Discussion

A significant strength of our study is the relatively large sample size of 184 patients. Of these, 129 did not meet the criteria for PCI based on ST elevation and symptom timing. Among 55 who met the criteria for PCI. The inclusion of patients with varying degrees of ST elevation and time delays of presentation further strengthens the generalizability of our findings. In addition, evaluating the measurements of ST-elevation across multiple leads adds comprehensiveness to understanding Spandan's performance in different anatomical regions of the heart.

### Principle finding

The BA plot of the analysis in this study confirmed that ST-segment elevation measurements obtained from the Spandan Pro smartphone-based ECG device are closely comparable to those obtained from the gold-standard 12-lead ECG. Analysis of all the standard leads—V1 to V6, I, II, III, aVL, and aVF—showed very small mean differences ranging from  −0.08 mV to 0.05 mV, showing no significant bias between the two systems. The 95% LOA, though differing slightly among leads, was within clinically acceptable levels. This tight range of differences suggests that, in the vast majority of cases, Spandan Pro and the gold-standard ECG produce nearly identical measurements. The BA plots provide further evidence for this interpretation, with random scatter around the mean difference line but no significant systematic bias. These results affirm that the Spandan Pro algorithm delivers robust and precise ST-elevation measurements in all leads and can be used interchangeably with the reference standard ECG for clinical decision-making, such as for guiding PCI, without losing diagnostic accuracy.

### Comparison with existing literature

The findings from previous studies showed the utility of portable ECG devices in detecting STEMI. It was seen that the prehospital ECG significantly decreased the door-to-balloon time, which, in turn, led to a better mortality level in cases of STEMI. Spaich et al. demonstrated that devices like CardioSecur are sensitive and specific for STEMI detection as Gold standard 12-lead ECGs in an ambulance environment [15]. Jung S et al., noted that the Alivecor Kardia Mobile 6L can diagnose STEMI effectively [19] and Avila CO, confirmed that the Apple Watch can reliably detect Cardiac Ischemia [11]. For this reason, the outcomes of this study suggest further validation of the utility of smartphone-based ECG devices to detect STEMI and are in line with increasing evidence supporting the integration of smartphone-based devices into routine cardiac care. This study is unique in that it appears to evaluate the feasibility, affordability, and portability of the Spandan Pro device in routine cardiac care.

### Limitations

Despite the growing popularity of smartphone-based ECG devices for detecting arrhythmia, several limits must be acknowledged. In this study, cases of ECG reports with baseline wandering or other artifacts were excluded. This exclusion may limit the generalizability of the findings to real clinical scenarios where such problems often occur. There are several limitations related to the usage of smartphone-based ECG devices. Factors related to the user include incorrect electrode placement, improper operation, and motion artifacts, which can degrade signal quality and provide inaccurate results. Technical issues like battery drain in the smartphone or connectivity issues may also impair the reliability of results. In addition, patients with baseline conduction abnormalities, comorbidities that make ECG morphology change, or those with implanted cardioverter defibrillators can yield pacing artifacts or modified QRS complexes that are difficult to interpret. In addition, the 5–6-h interval between the smartphone ECG and gold standard 12-lead ECG recordings may introduce minor clinical variation with a possible impact on results comparability.

### Clinical implication

Spandan Pro offers a well-portable, affordable alternative to the Gold standard 12-lead ECG for STEMI diagnosis. It takes almost one-fifth the cost (5:1 ratio) of a gold standard ECG machine, which is an estimated 80% lower price. In addition to affordability, it can potentially save overall treatment costs by enabling early diagnosis and early intervention. It is user-friendly and accurate enough to be suitable for prehospital, resource-limited, and emergency use, potentially introducing some quality improvements into expedient STEMI diagnosis and thereby bettering the outcome.

Smartphone-based ECGs are integrated into current EMS and triage through technology that enables real-time transmission, remote interpretation, and prehospital alerts. This integration dramatically accelerates diagnosis and treatment for cardiac emergencies, especially ST-elevation MI.

## Conclusion

The study demonstrated that the Spandan Pro smartphone-based ECG device is a reliable alternative to the gold-standard 12-lead ECG for diagnosing STEMI. Using BA analysis, the study found minimal bias in the ST-elevation measurements between Spandan Pro and the Gold standard ECG across various leads, particularly leads I, II, and V5, where near-perfect agreement was observed. The 95% LOA in all leads are within clinically acceptable limits, which makes Spandan Pro substitutable with the conventional ECG in most cases.

Its portability and user-friendliness make the device especially important for the early diagnosis of patients in prehospital or resource-constrained settings in which timely detection of STEMI is crucial for better patient outcomes. The Spandan Pro device could contribute to the rapid decisions in cases of STEMI, comparable to the gold standard ECG, by allowing faster intervention and also better adherence to PCI guidelines.

In summary, the high diagnostic accuracy values combined with close agreement in the BA analysis place the Spandan Pro device as viable and effective for STEMI detection in general, which in turn may also give potential improvements in both prehospital care and emergency room settings.

## Data Availability

No datasets were generated or analyzed during the current study.

## Abbreviations

ECG: Electrocardiogram
STEMI: ST-elevation Myocardial Infarction
NSTEMI: Non-ST-elevation Myocardial Infarction
PCI: Percutaneous Coronary Intervention
CAD: Coronary Artery Disease
MI: Myocardial Infarction
BA: Bland–Altman
LOA: Limits of Agreement
